# Health Benefits and Molecular Mechanisms of Resveratrol: A Narrative Review

**DOI:** 10.3390/foods9030340

**Published:** 2020-03-14

**Authors:** Xiao Meng, Jing Zhou, Cai-Ning Zhao, Ren-You Gan, Hua-Bin Li

**Affiliations:** 1 Guangdong Provincial Key Laboratory of Food, Nutrition and Health, Department of Nutrition, School of Public Health, Sun Yat-Sen University, Guangzhou 510080, China; mengx7@mail2.sysu.edu.cn (X.M.); lihuabin@mail.sysu.edu.cn (H.-B.L.); 2 School of Public Health, Hainan Medical University, Haikou 571199, China; hy0208035@hainmc.edu.cn; 3 Department of Clinical Oncology, Li Ka Shing Faculty of Medicine, The University of Hong Kong, Hong Kong 999077, China; zhaocn@hku.hk; 4 Research Center for Plants and Human Health, Institute of Urban Agriculture, Chinese Academy of Agricultural Sciences, Chengdu 610213, China

**Keywords:** resveratrol, bioactivities, anticancer, anti-obesity, antidiabetes, molecular mechanisms

## Abstract

Resveratrol is a bioactive compound in many foods. Since its anticancer activity was reported in 1997, its health benefits have been intensively investigated. Resveratrol has antioxidant, anti-inflammatory, immunomodulatory, glucose and lipid regulatory, neuroprotective, and cardiovascular protective effects, therefore, can protect against diverse chronic diseases, such as cardiovascular diseases (CVDs), cancer, liver diseases, obesity, diabetes, Alzheimer’s disease, and Parkinson’s disease. This review summarizes the main findings of resveratrol-related health benefits in recent epidemiological surveys, experimental studies, and clinical trials, highlighting its related molecular mechanisms. Resveratrol, therefore, has been regarded as a potent candidate for the development of nutraceuticals and pharmaceuticals to prevent and treat certain chronic diseases.

## 1. Introduction

Many foodstuffs and their bioactive components are beneficial to certain diseases including cardiovascular diseases (CVDs) and cancer [1,2]. Resveratrol is a polyphenol, which naturally occurs in numerous foods, such as blueberries and peanuts, as well as grapes and their derived products like red wine [3]. Since resveratrol was reported to possess strong anticancer properties in tumor initiation, promotion, and progression stages in the well-regarded journal *Science* in 1997 [4], its bioactivities and health benefits have been intensively investigated. Many epidemiologic studies demonstrated that resveratrol is effective in the prevention of some diseases, such as CVDs and cancer [5,6]. Additionally, numerous experimental studies have illustrated that resveratrol is beneficial to a broad range of diseases, including CVDs, diabetes, obesity, cancer, liver diseases, Alzheimer’s disease, and Parkinson’s disease through redox/inflammatory/immune signaling pathways as well as the interplay between lipid and glucose metabolism [7,8,9,10,11,12,13,14]. Driven by the promising results from experimental research, many clinical trials have also elicited the efficacy of resveratrol against certain diseases [15,16]. 

This review summarizes the main findings regarding the bioactivities and health impacts of resveratrol, based on systematically searching English literature from Web of Science Core Collection and PubMed in the last five years, using the key word “resveratrol”. The literature was categorized into epidemiological surveys, experimental studies, and clinical trials. In particular, this review highlights the health benefits of resveratrol on chronic diseases including CVDs, cancer, liver diseases, obesity, diabetes, Alzheimer’s disease, and Parkinson’s disease, and its related molecular mechanisms. We hope that this narrative review paper can provide updated information about resveratrol and can attract more attention to its health benefits.

## 2. Observational Studies 

Since resveratrol was illustrated to be one of the key factors in wine contributing towards the French paradox (high intake of saturated fat but low mortality from coronary heart disease), it has attracted overwhelming interest worldwide, and many epidemiological studies have investigated the relationship between resveratrol consumption and human health [17,18,19,20]. Specifically, dietary patterns rich in resveratrol were demonstrated to significantly reduce all-cause mortality [21,22]. Resveratrol also showed its potential to improve CVD risk factors, presenting significantly decreased fasting blood glucose, triglycerides (TGs), and heart rate [6]. In addition, a case-control study reported a significant inverse association between resveratrol from grapes (but not from wine) and breast cancer risk [17]. Furthermore, a lower risk of esophageal cancer was linked with higher resveratrol intake [5]. However, some null results or even harmful effects on health have also been reported. For instance, in a cross-sectional study in the Iranian population, resveratrol intake (top quantile, 0.054 mg/day and more) was positively associated with high blood pressure (hazard ratios, HR: 1.52; 95% confidence intervals, 95% CI: 1.02–2.27), without an significant association with waist circumference, TG, high-density lipoprotein (HDL), blood glucose, and metabolic syndrome [19]. Similar null outcomes were found in other studies [18,23]. In fact, the findings from different epidemiological studies are often inconsistent, because their validity depends on many factors like the study design, sample size, resveratrol dose, follow-up duration, as well as the participants’ race, health status, eating patterns, and their food preference (Table 1). Nevertheless, the positive results support further exploration of what other health effects resveratrol might provide and how it achieves them.

## 3. Experimental Studies

Given the observed benefits based on resveratrol consumption, a great deal of research has explored more health outcomes of resveratrol as well as the underlying molecular mechanisms.

### 3.1. Antioxidative Activities

**Figure 1:**
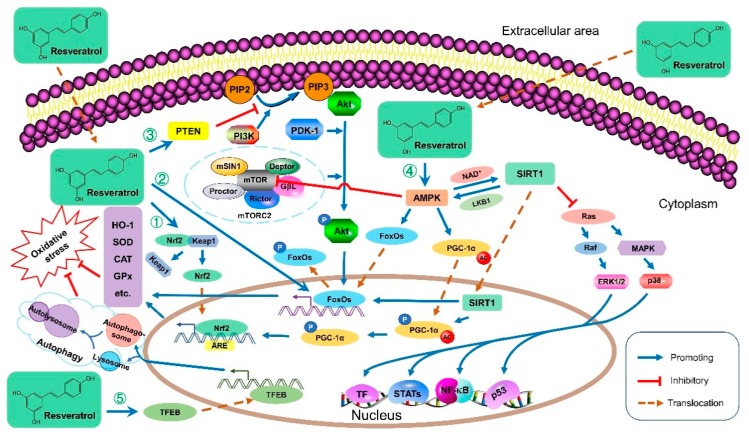
The antioxidant molecular mechanisms of resveratrol. ① Resveratrol unanchors Nrf2 in the cytoplasm, disrupting its Keap1-dependent ubiquitination and degradation. The built-up Nrf2 translocates into the nucleus, binds to ARE, and initiates the transcription of many antioxidative genes such as SOD and CAT to reduce oxidative stress. ② Resveratrol promotes the transcriptional functions of FoxOs in the nucleus to facilitate the transcription of many antioxidative genes like HO-1, contributing to the reduction of oxidative stress. ③ Resveratrol upregulated PTEN, a major antagonist of PI3K, blocking the Akt activation. Consequently, the activated Akt reduces, leading to decreased FoxOs phosphorylation. Therefore, less *p*-FoxOs translocate from the nucleus to the cytoplasm, and more FoxOs remain in the nucleus to act as transcriptional factors. ④ Resveratrol activates AMPK to maintain the structural stability of FoxOs, facilitate its translocation, and accomplish its transcriptional function. In addition, the activated AMPK phosphorylates PGC-1α, which can translocate into the nucleus, and be deacetylated by SIRT1. Then PGC-1α promotes Nrf2, leading to increased antioxidative gene expression and then reduced oxidative stress. Resveratrol activates AMPK, leading to SIRT1 activation, which inhibits MAPK signaling pathways and results in autophagy. ⑤ Resveratrol induces autophagy by activating TFEB, which promotes the formation of autophagosome and lysosome as well as their fusion into an autolysosome, leading to reduced oxidative stress. Abbreviations: AC, acetyl; Akt, protein kinase B; AMPK, AMP-activated protein kinase; ARE, antioxidant response element; CAT, catalase; ERK, extracellular signal-regulated kinase; FoxO, forkhead box protein O; GPx, glutathione peroxidase; GβL, G protein β subunit-like; HO-1, heme oxygenase 1; Keap1, Kelch-like ECH-associated protein 1; LKB1, liver kinase B1; MAP2K, mitogen-activated protein kinase kinase; MAPK, mitogen-activated protein kinase; mSIN1, mammalian stress-activated protein kinase interacting protein 1; mTOR, mammalian target of rapamycin; mTORC2, mTOR Complex 2; NAD, nicotinamide adenine dinucleotide; NF-κB, nuclear factor kappa-light-chain-enhancer of activated B cells; Nrf2, nuclear factor (erythroid-derived 2)-like 2; P, phosphorylation; p53, phosphoprotein p53; PDK1, phosphoinositide dependent kinase 1; PGC-1α, peroxisome proliferator-activated receptor gamma coactivator 1α; PI3K, phosphatidylinositol 3-kinase; PIP2, phosphatidylinositol 4,5-bisphosphate; PIP3, phosphatidylinositol-3,4,5-trisphosphate; PTEN, phosphatase and tensin homolog; Rictor, the rapamycin-insensitive companion of mTOR; SIRT1, sirtuin 1; SOD, superoxide dismutase; STAT, signal transducer and activator of transcription; TF, transcription factor; TFEB, transcription factor EB.

Resveratrol has shown strong antioxidant properties in many studies [24,25]. Resveratrol protects against oxidative stress, one of the primary contributors to many diseases, through various redox-associated molecular pathways (Figure 1 and Table 2). For instance, resveratrol upregulated the phosphatase and tensin homolog (PTEN), which decreased Akt phosphorylation, leading to an upregulation of antioxidant enzyme mRNA levels such as catalase (CAT) and superoxide dismutase (SOD) [26]. Resveratrol could also improve the antioxidant defense system by modulating antioxidant enzymes through downregulation of extracellular signal-regulated kinase (ERK) activated by reactive oxygen species (ROS) [27]. Meanwhile, resveratrol reduced the ischemia-reperfusion injury-induced oxidative stress by inhibiting the activation of the p38 mitogen-activated protein kinase (MAPK) pathway, thus the levels of antioxidants like glutathione (GSH) increased, and free radicals were directly scavenged [28]. Furthermore, resveratrol activated adenosine monophosphate (AMP)-activated protein kinase (AMPK) to maintain the structural stability of forkhead box O1 (FoxO1), facilitate its translocation, and accomplish its transcriptional function [25]. Moreover, resveratrol was demonstrated to improve systemic oxidative and nitrosative stress by activating AMPK, then sirtuin 1 (SIRT1) and the nuclear factor erythroid-2-related factor 2 (Nrf2) associated antioxidant defense pathways, as Nrf2 acts as the master regulator of numerous genes encoding antioxidants and phase II-detoxifying enzymes and molecules [29,30]. Additionally, resveratrol exhibited antioxidant bioactivities by regulating antioxidant gene expression via the Kelch-like ECH-associated protein 1 (Keap1)/Nrf2 pathway and SIRT1 [31]. Recently, resveratrol was reported to attenuate oxidative injury owing to the induced autophagy via the AMPK-mediated inhibition of mammalian target of rapamycin (mTOR) signaling or via the activation of transcription factor EB (TFEB), which promoted the formation of autophagosomes and lysosomes as well as their fusion into an autolysosome [32,33]. Generally, resveratrol protects against oxidative stress mainly by (i) reducing ROS/reactive nitrogen species (RNS) generation; (ii) directly scavenging free radicals; (iii) improving endogenous antioxidant enzymes (e.g., SOD, CAT, and GSH); (iv) promoting antioxidant molecules and the expression of related genes involved in mitochondrial energy biogenesis, mainly through AMPK/SIRT1/Nrf2, ERK/p38 MAPK, and PTEN/Akt signaling pathways; (v) inducing autophagy via mTOR-dependent or TFEB-dependent pathway.

### 3.2. Anti-Inflammatory Activities

Resveratrol has been illustrated to have potent anti-inflammatory activities in many studies (Table 2) [34,35,36,37]. Resveratrol protected from inflammation not only by inhibiting the production of pro-inflammatory cytokines such as tumor necrosis factor α (TNF-α) and interleukin-1β (IL-1β), but also by inducing anti-inflammatory heme oxygenase-1 (HO-1) in RAW264.7 macrophages [38]. Additionally, resveratrol suppressed IL-6 transcription, modulating the inflammatory responses as an estrogen receptor α (ERα) ligand mediated by SIRT1, which functions as an ER coregulator [39]. Resveratrol could also inhibit nuclear factor kappa-light-chain-enhancer of activated B cells (NF-κB) signaling independent of SIRT1 [40]. Moreover, resveratrol attenuated inflammation by downregulating high mobility group box 1 (HMGB1) as well as suppressing NF-κB and Janus kinase (JAK)/signal transducer and activator of transcription (STAT) signaling pathways [41,42]. In addition, both in vitro and in vivo, the anti-inflammatory effects of resveratrol were associated with its inhibition of the toll-like receptor 4 (TLR4)/NF-κB signaling cascade [43,44]. Furthermore, resveratrol attenuated inflammation by inhibiting the activation of NACHT, LRR, and PYD domains-containing protein 3 (NALP3) inflammasome and inducing autophagy via SIRT1 upregulation and AMPK activation [34,35]. In a study on osteoarthritis, resveratrol interrupted an inflammatory amplification loop [45]. Specifically, the resveratrol-induced NF-κB inhibition resulted in decreased IL-6 secretion, leading to suppressed signal transducer and activator of transcription 3 (STAT3) activation in macrophages. Since STAT3 was responsible for the positive regulation of IL-6 secretion, inhibition of STAT3 made IL-6 levels even lower. Resveratrol could also block ERK1/2 activation, consequently upregulating MyD88 Short, a negative regulator of inflammation [36]. Altogether, resveratrol was able to regulate the pro- and anti-inflammatory cytokines and chemokines to protect against inflammation, mainly by upregulating SIRT1, suppressing NF-κB, and the associated cascades, as well as inhibiting NALP3 inflammasome activation.

### 3.3. Immunomodulating Effects

Resveratrol has exerted immunomodulating effects in various studies (Table 2) [46,47,48]. Resveratrol modulates immune response against pathogens like viruses, bacteria, and some toxic materials. For instance, resveratrol upregulated immune responses and reduced immunocyte apoptosis in chickens receiving conventional vaccinations and improved the growth of young chickens [49]. Resveratrol also reduced the activity of respiratory syncytial virus and inhibited the toll/IL-1 receptor domain-containing adaptor inducing β interferon (TRIF) expression through upregulating sterile α and armadillo motif protein (SARM) [50]. In addition, resveratrol prevented enterovirus 71 (EV71) replication and reduced the virus-induced elevated IL-6 and TNF-α secretion in rhabdosarcoma cells via suppressing IκB kinase (IKK)/NF-κB signaling pathway [48]. Moreover, resveratrol inhibited human rhinoviruses-16 replication and normalized virus-induced IL-6, IL-8 and regulated on activation normal T cell expressed and secreted (RANTES) as well as the expression of intercellular adhesion molecule-1 (ICAM-1) [47]. Additionally, resveratrol maintained the immune function in rotavirus-infected piglets, resulting in attenuated diarrhea and inflammation [51]. Moreover, resveratrol triggered an immune response to protect against non-typeable *Haemophilus influenzae* (a respiratory bacterium) without developing resistance in vitro [46]. Resveratrol also decreased bacterial viability and reduced infectious airway inflammation without noticeable host toxicity in vivo [46,52]. In addition, resveratrol showed immunomodulatory properties via reducing bacterial and inflammatory biomarkers in lipopolysaccharides (LPS)-challenged primary Atlantic salmon macrophages [52]. Resveratrol also modulated immunity caused by some toxic materials like concanavalin A (Con A), showing upregulation of SIRT1 and reduction of cytokines such as TNF-α, interferon γ (IFN-γ), IL-6, and monocyte chemoattractant protein-1 (MCP-1) [53]. Interestingly, resveratrol was found to strongly enhance immune activity in immunosuppressive mice, showing a bidirectional regulatory effect on immunity [54]. Specifically, resveratrol improved spleen lymphocyte proliferation, enhanced the function of peritoneal macrophages, and increased the CD4^+^ cells in peripheral blood. Furthermore, some cytokines in the serum were upregulated, such as IL-1α/β, IL-2, and TNF-α. Based on a statistical analysis of human microarray data, a recent study revealed that resveratrol regulated many immune response pathways including peroxisome proliferator-activated receptor α (PPAR-α)/retinoid X receptor α (RXRα) activation, IL-10 signaling, natural killer cell signaling, leucocyte extravasation signaling, and IL-6 signaling [55]. Recently, it was revealed that resveratrol could suppress the aryl hydrocarbon receptor (AhR) pathway, resulting in the reversal of imbalanced Th17/Treg, the main characteristic of immune thrombocytopenic purpura [56].In short, resveratrol could modulate both cellular and humoral immunity to reduce replication and the viability of pathogens, and bidirectionally regulate the related cytokine/chemokine production through the canonical immune response pathways as mentioned above.

### 3.4. Cardiovascular Diseases

Resveratrol has been reported to protect against CVDs in certain research (Table 2) [57,58,59]. Resveratrol prevented the pathological progression of hypertension, a major risk factor of CVDs, through Nrf2 activation, owing to its antioxidant and anti-inflammatory capacity [60]. Resveratrol could also lower blood pressure in hypertensive mice by inducing oxidative activation of cyclic guanosine monophosphate (cGMP)-dependent protein kinase 1α (PKG1α) [61]. Atherosclerosis is another main contributor to CVDs. Resveratrol was able to block atherosclerotic plaque progression by acting against pro-atherogenic oxysterol signaling in M1 (inflammation-encouraging) and M2 (inflammation-decreasing) macrophages [57]. Meanwhile, resveratrol prevented the activation of inflammasome, a contributor to the vascular inflammatory injury and atherosclerosis, via downregulating NF-κB p65 and p38 MAPK expression, and upregulating SIRT1 expression [62]. In addition, resveratrol ameliorated atherosclerosis partially through restoring intracellular GSH via AMPK-α activation, resulting in inhibited monocyte differentiation and reduced pro-inflammatory cytokine production [59]. Moreover, resveratrol regulated the band 4.1, ezrin, radixin, and moesin (FERM)-kinase and Nrf2 interaction, leading to decreased expression of ICAM-1 and then the inhibition of monocyte adhesion [63]. Resveratrol also exhibited antithrombotic effects via decreasing the tissue factors like TNF-α, and such action could be facilitated by aortic endothelial cells that could deconjugate resveratrol metabolites to free resveratrol [64]. Furthermore, one of the atherosclerosis consequences, high fat/sucrose diet-induced central arterial wall stiffening, was improved by resveratrol based on its protective activities against oxidative stress and inflammation [7]. Resveratrol also effectively prevented CVD by improving the cardiac and vascular autonomic function, protecting the erythrocytes via interacting with hemoglobin and reducing heme-iron oxidation [65,66]. In a heart failure model, resveratrol mitigated atrial fibrillation by upregulating PI3K and endothelial NOS (eNOS) [8]. In summary, the cardiovascular protective effects of resveratrol mainly depend on the capabilities of reducing oxidative stress and alleviating inflammation through Nrf2 and/or SIRT1 activation, PI3K/eNOS upregulation, and NF-κB downregulation.

### 3.5. Cancers

Resveratrol has exhibited protective impacts on various cancers, like colorectal cancer, lung cancer, breast cancer, prostate cancer, ovarian cancer, cervical cancer, liver cancer, and gastric cancer (Table 2) [14,67,68,69,70,71]. For instance, resveratrol was reported to inhibit the formation and growth of colorectal cancer by downregulating oncogenic KRAS expression [68]. Resveratrol also prevented tumorigenesis and progression of non-small cell lung cancer (NSCLC) cells by interrupting glycolysis via inhibition of hexokinase II expression, which was mediated by downregulation of the epidermal growth factor receptor (EGFR)/Akt/ERK1/2 signaling pathway [69]. Moreover, resveratrol showed pro-apoptotic/anti-proliferative effects in LNCaP cells (human prostate adenocarcinoma cells) through inducing the expression of cyclooxygenase (COX)-2, promoting ERK1/2 activation, and facilitating p53-dependent anti-proliferation gene expression [14]. In addition, resveratrol could decrease the efficiency of ovarian cancer cells adhering to peritoneal mesothelium in vitro by downregulating the production of α5β1 integrins and upregulating the release of soluble hyaluronic acid [70]. Resveratrol was also reported to inhibit the expression of phospholipid scramblase 1 (PLSCR1), leading to the growth inhibition of HeLa cells [71]. Furthermore, resveratrol showed proliferation-inhibitory and apoptosis-inducing effects in HepG2 cells by activating caspase-3 and caspase-9, upregulating the Bax/Bcl-2 ratio, and inducing p53 expression [72]. Resveratrol also inhibited the invasion and migration of human gastric cancer cells by blocking the epithelial-to-mesenchymal transition, which was mediated by metastasis-associated lung adenocarcinoma transcript 1 (MALAT1) [73]. Additionally, resveratrol protected against breast cancer metastasis by promoting antitumor immune responses via blunting STAT3, leading to inhibited generation and function of tumor-evoked regulatory B cells (tBregs) as well as decreased production of transforming growth factor β (TGF-β) (a downstream target of STAT3), which was required by the tBregs to convert resting CD4^+^ T cells to the metastasis-promoting FoxP3^+^ regulatory T cells (Tregs) [67]. Although some research showed that resveratrol may be beneficial in breast cancer chemoprevention due to its non-estrogen function [74], different doses of resveratrol showed controversial effects due to its interaction with estrogens, which induce cellular proliferation and play a key role in breast cancer development and progression. Specifically, it was found that high concentrations of resveratrol could inhibit the proliferation of estrogen receptor alpha positive (ERα+) breast cancer, while low concentrations increased the growth of ERα+ cells [75,76]. It was reported that at low concentrations, resveratrol not only bound to ERs due to its structural similarity with E2, but also increased the formation of estrogen precursor steroids and inhibited the inactivation of active steroids, resulting in elevated active estrogen levels, leading to breast cancer cell growth and progression [77,78]. However, “more resveratrol is better” was challenged in some cases [79]. For instance, in terms of colorectal cancer chemoprevention, lower doses of resveratrol from dietary exposures exerted a better efficacy than high doses (200 times higher previously used in phase I clinical trials) due to its pro-oxidant activity at high doses and AMPK signaling upregulation.

Collectively, resveratrol has shown its anticancer bioactivities by impairing glycolysis, inhibiting cancer cell growth and proliferation, inducing apoptosis, promoting antitumor immune responses, and preventing adhesion, migration and invasion of cancer cells by modulating related molecules and gene expression through various signaling pathways. Of note, different doses may lead to very different effects, which sometimes could be opposite. Therefore, consideration regarding doses and matrix should be paid more attention in future studies.

### 3.6. Liver Diseases

Resveratrol has shown its protective impacts on several liver diseases in some studies (Table 2) [9,80,81,82]. Specifically, resveratrol alleviated non-alcoholic fatty liver disease (NAFLD) by upregulating the low-density lipoprotein receptor (LDLR) and scavenger receptor class B type I (SRB1) gene expressions in the liver [83], or by regulating autophagy and decreasing the activity of NF-κB, resulting from restoring its inhibitor, nuclear factor of kappa light polypeptide gene enhancer in B-cells inhibitor α (IκBα) [84]. Resveratrol also improved high-fat diet (HFD)-induced fatty liver by downregulating adipose differentiation-related proteins and increasing the numbers of CD68^+^ Kupffer cells [9]. As for chemical-induced liver diseases, resveratrol could markedly restore the morphology and function of alcohol-injured liver through inducing autophagy, or downregulating hypoxia-inducible factor 1α (HIF-1α) expression [85,86]. In addition, resveratrol ameliorated CCl_4_-induced liver injury by blocking the Notch signaling pathway [82]. Furthermore, resveratrol attenuated N′-Nitrosodimethylamine-induced hepatic fibrosis by restoring the architecture and normalizing collagen deposition, mainly due to its antioxidative activities and downregulation of smooth muscle actin (α-SMA), which suppressed hepatic stellate cell activation [80,81]. Moreover, resveratrol pretreatment mitigated liver cirrhosis by improving the homing of bone marrow-derived mesenchymal stem cells [87]. In summary, resveratrol could improve NAFLD, chemical-induced liver injuries, fibrosis, and cirrhosis by modulating redox status, regulating lipid metabolism, ameliorating inflammation, and inducing autophagy with various cytokines, chemokines, and transcription factors involved. 

### 3.7. Diabetes

Resveratrol has been elicited to attenuate diabetes and its relevant complications in many studies (Table 2) [88,89,90,91]. Resveratrol was observed to significantly reduce blood glucose levels, plasma lipids, and free fatty acids in diabetic mice, and it inhibited the expression of inflammatory mediators (e.g., ICAM-1, vascular cell adhesion molecule-1, and MCP-1) both in the aorta and in the blood, by inhibiting the NF-κB pathway [92]. In addition, resveratrol could relieve diabetes via increasing insulin action and glucose utilization due to visfatin expression restoration, SIRT1 activation, and glucose transporter modulation [89]. Moreover, resveratrol increased glucose uptake to improve insulin resistance in the muscle by decreasing diacylglycerol (DAG) accumulation and protein kinase C θ (PKC-θ) translocation, and preventing lipolysis under the condition of adipose hypoxia, because resveratrol could preserve phosphodiesterase 3B expression (PDE 3B) to downregulate cyclic adenosine monophosphate (cAMP), leading to the inhibition of protein kinase A (PKA)/hormone-sensitive lipase (HSL) activation [90]. Moreover, resveratrol showed protective effects on adipose tissue in diabetic mice by preventing ROS-mediated mitochondrial fission via AMPK-dependent upregulation of Drp1 phosphorylation, and by blocking the activation of NALP3 inflammasome via inhibition of endoplasmic reticulum stress (ERS) [93]. Resveratrol also protected against diabetic complications such as myocardial fibrosis, diabetic nephropathy, and erectile dysfunction [11,88,94]. Furthermore, maternal resveratrol administration to the rats was evidenced to prevent the offspring’s glucose intolerance and islet dysfunction, which were associated with gestational diabetes [91]. In summary, resveratrol could effectively regulate glucose metabolism, improve insulin resistance, improve diabetic complications, and restore the function of multiple systems via modulating SIRT1/NF-κB/AMPK signaling pathways and some associated molecules like NALP3 inflammasome, as well as the expressions of relevant genes.

### 3.8. Obesity

Obesity has become a severe health issue globally. Resveratrol significantly decreased the body weight and fat mass in mice with HFD-induced obesity, showing reduced leptin and lipids in plasma, modulated metabolism of glucose and insulin, and restored immune dysfunction, via the activation of PI3K/SIRT1 and Nrf2 signaling pathways, and the inhibition of transcriptional regulators (e.g., EP300 gene), which are involved in the differentiation of adipocytes as well as lipid storage and metabolism [95,96]. Moreover, besides a significant dose-dependent decrease of weight gain and lipid deposition in the liver and adipose tissues of HFD-induced obese mice, low concentrations of resveratrol (1–10 μM) suppressed adipogenic differentiation in pre-adipocytes, downregulated the expression of peroxisome proliferator-activated receptor γ (PPAR-γ) and perilipin protein in differentiated adipocytes, and inhibited TNF-α-induced lipolysis in mature adipocytes [97]. Additionally, resveratrol prevented against obesity through markedly enhancing the catecholamine production, accompanied by suppressing the pro-inflammatory M1 macrophages and activating anti-inflammatory M2 macrophages in white adipose tissue, which play a pivotal role in the trans-differentiation of white adipocytes into beige adipocytes [98]. Furthermore, resveratrol administrated to the pregnant and lactating mice led to promoted white adipose browning and thermogenesis in the male descendants, and these health benefits persisted and prevented obesity in their future life [99]. In addition, resveratrol protected against sarcopenic obesity by improving mitochondrial function and reducing oxidative stress through the PKA/liver kinase B1 (LKB1)/AMPK pathway [100]. Resveratrol also showed positive impacts on obesity-related complications, such as reproductive dysfunction like infertility and endocrine disorders [101,102]. To summarize, resveratrol has been illustrated to decrease body weight, regulate lipid deposition, modulate adipocyte gene expression, and promote white adipose browning, via PI3K/SIRT1, Nrf2, PPAR-γ, TNF-α, and PKA/LKB1/AMPK signaling pathways (Table 2).

### 3.9. Alzheimer’s Disease and Parkinson’s Disease

Alzheimer’s disease and Parkinson’s disease are neurodegenerative disorders, seriously decreasing life quality, while resveratrol may have the potential to improve these diseases. For instance, resveratrol inhibited the aggregation of amyloid β (Aβ), a key factor in Alzheimer’s disease, by modulating specific proteins such as ubiquitin-like protein (UBL)/X-box binding protein 1 (XBP-1) involved in proteostasis [103]. Furthermore, resveratrol prevented memory loss in Alzheimer’s disease by decreasing elevated levels of mitochondrial complex IV protein in the mouse brain via the activation of SIRT1 and AMPK pathways [104,105]. In terms of Parkinson’s disease, resveratrol ameliorated ERS by downregulating the gene expression of C/EBP homologous protein (CHOP) and glucose-regulated protein 78 (GRP78), inhibiting caspase-3 activity in the rat brain, and ameliorating oxidative damage via suppressing xanthine oxidase activity and protein carbonyl formation as well as activating the glutathione peroxidase and Nrf2 signaling pathway [10]. Resveratrol also alleviated Parkinson’s disease through elevating miR-214 expression, leading to decreased mRNA expression of α-synuclein [106]. Taken together, resveratrol ameliorated Alzheimer’s disease and Parkinson’s disease by activating the SIRT1, AMPK, and Nrf2 signaling pathways and modulating the associated gene expressions (Table 2).

### 3.10. Sex-Dependent Effects of Resveratrol

Acting as an estrogen agonist, resveratrol showed sex-dependent effects on some diseases, which causes increasing concerns (Table 2). CVD risk increases with increasing age, gradually in men while disproportionately in women, and such a lower risk was in association with estrogen’s cardioprotective properties [107,108]. In a recent in vivo study, sex differences were observed in rats with surgically-induced myocardial infarction (MI) due to the resveratrol treatment (2.5 mg/kg/d) [109]. Superior improvements were observed in females in terms of interventricular septal wall dimension at systole (IVSDs), end-systolic volume (ESV), ejection fraction (EF), fractional shortening (FS), and isovolumic relaxation time (IVRT), among which IVRT was purely sex-dependent. In another study, long-term resveratrol treatment in rats (50 mg/L in drinking water, 21 days) increased the relaxations to estrogen in aortae, more potently in males, probably due to the effects of resveratrol on promoting nitric oxide and/or suppressing superoxides [110]. Alongside this, it was revealed that resveratrol (20 mg/kg) significantly increased dopamine transporter (DAT) in the striatum in female but not in male mice [111]. The in vitro study in the same research indicated that resveratrol upregulated DAT in the dopaminergic cells by inducing its gene transcription. Additionally, sex differences in the effect of resveratrol were also found in a mouse model of dextran sulfate sodium-induced colitis [112]. Adverse effects were observed in females but not in males, regarding weight loss, stool consistency, and discomfort. Such results indicate that special attention should be paid to the application of resveratrol, a phytoestrogen, which can interact with hormone receptors and result in sex-dependent effects that can be beneficial or harmful.

**Table 2:** Bioactivities and potential mechanisms of resveratrol from experimental studies.

Study Type	Subject	Dose	Main Findings	Ref.
*Antioxidative activities*				
In vitro	HepG2 cells	0–100 μM	Dose-dependently increasing antioxidant effects by enhancing SIRT2’s activity to deacetylate Prx1	[24]
In vitro	HepG2, C2C12, and HEK293 cells	10, 25 μM	Activating AMPK to maintain the structural stability of FoxO1	[25]
In vitro	MCF-7 cells	1 nM, 0.02 μM, 0.1 μM, 0.5 μM, 1.5 μM	Upregulating PTEN (except at the highest dose, 1.5 μM), which decreased Akt phosphorylation, leading to an upregulation of antioxidant enzyme mRNA levels such as CAT and SOD	[26]
In vivo	Rats	20 mg kg/b.w./day	Improving the antioxidant defense system by modulating antioxidant enzymes through downregulation of ERK activated by ROS	[27]
In vivo	Rats	10 mg/kg b.w.	Reducing the ischemia-reperfusion injury-induced oxidative stress by inhibiting the activation of p38 MAPK pathway to increase antioxidants like GSH and scavenge free radicals	[28]
In vivo	Rats	5, 10 mg/kg	Activating SIRT1 to scavenge ROS	[29]
In vivo	Mice	15, 30, 60 mg/kg	Activating AMPK, SIRT1, and Nrf2 associated antioxidant defense pathways to improve systemic oxidative and nitrosative stress	[30]
In vivo	Sows	300 mg/kg	Regulating antioxidant gene expression via Keap1/Nrf2 pathway and SIRT1	[31]
In vitro	HUVECs	10 μM	Inducing autophagy via the activation of TFEB	[32]
In vitro	HEK293 cells or HEK293T	5 μg/mL	Inducing autophagy via the AMPK-mediated inhibition of mTOR signaling	[33]
*Anti-inflammatory activities*				
In vivo	Mice	8 mg/kg/day	Inhibiting the activation of NALP3 inflammasome and inducing autophagy via SIRT1 upregulation	[34]
In vitro	J774 mouse macrophages, Mouse bone-marrow cells	0.5–100 μM	Inhibiting the activation of NALP3 inflammasome	[35]
In vitro; In vivo	BEAS-2B cells, Mice	25 μM, 20 mg/kg	Inducing NF-κB inhibition, decreasing IL-6 secretion, suppressing STAT3 activation, blocking ERK1/2 activation, and upregulating MyD88 Short	[36]
In vitro	RAW264.7 macrophages	0–20 μM	Inhibiting the production of pro-inflammatory cytokines, such as TNF-α and IL-1β, but also by inducing anti-inflammatory HO-1	[38]
In vitro	RAW264.7 macrophages, MCF-7 cells	10 μM	Suppressing IL-6 transcription, modulating the inflammatory responses as an ERα ligand mediated by SIRT1.	[39]
In vitro	Mouse C2C12 myoblasts	20, 50, 100 μM	Inhibiting NF-κB signaling independent of SIRT1	[40]
In vitro	RAW264.7 macrophages	1, 5, 10, 20, 40 μM	Downregulating HMGB1 as well as suppressing NF-κB and JAK/STAT signaling pathways	[41]
In vitro	U937 monocytic cells	15, 30, 50 μM	Inhibiting NF-κB and JAK/STAT signaling pathways	[42]
In vitro In vivo	NRK-52E, Rat	100 μmol/mL, 0.23 μg/kg	Inhibiting TLR4/NF-κB signaling cascade	[43]
In vivo	Rats	30, 10 and 3 mg/kg,	Inhibiting TLR4/NF-κBp65/MAPKs signaling cascade	[44]
In vitro	Primary chondrocytes and macrophages	10, 25, 50, 100 μM	Interrupting an inflammatory amplification loop	[45]
*Immunomodulating effects*				
In vitro	A549 cells	56.25, 112.5 μg/mL	Triggering an immune response to protect against non-typeable *Haemophilus influenzae* without developing resistance	[46]
In vitro	H1HeLa cells, Human nasal epithelia	0–300 μM	Inhibiting human rhinoviruses-16 replication and normalized virus-induced IL-6, IL-8, and RANTES as well as the expression of ICAM-1	[47]
In vitro	Rhabdosarcoma cells	2.5–100 μg/mL	Preventing EV71 replication, reducing the virus-induced elevated IL-6 and TNF-α secretion via suppressing IKK/NF-κB signaling pathway	[48]
In vivo	Chickens	200, 400, 800 mg/kg	Reducing immunocyte apoptosis in chickens receiving conventional vaccinations, and improving the growth of young chickens	[49]
In vivo	Piglets	3, 10, 30 mg/kg/d	Maintaining the immune function and attenuating diarrhea and inflammation	[51]
In vitro	Atlantic salmon macrophages	10, 30, 50 μM	Reducing bacterial and inflammatory biomarkers in LPS-challenged primary Atlantic salmon macrophages	[52]
In vivo	Mice	30 mg/kg	Upregulating SIRT1 and reducing cytokines such as TNF-α, IFN-γ, IL-6, and MCP-1	[53]
In vivo	Mice	30 mg/kg	Enhancing immune activity in immunosuppressive mice, showing a bidirectional regulatory effect on immunity	[54]
In vitro	Human CD4+ T cells	10, 30, or 50 μM	Suppressing the AhR pathway, resulting in the reversal of imbalanced Th17/Treg	[56]
*Cardiovascular diseases*				
In vivo	Rhesus monkeys	80 mg/day (1st year), 480 mg/day (2nd year)	Improving central arterial wall stiffening based on its antioxidative and anti-inflammation	[7]
In vivo	Rabbits	2.5 mg/kg	Mitigating atrial fibrillation by upregulating PI3K/AKT/eNOS	[8]
In vitro	Peripheral blood mononuclear cells	3–80 μM	Blocking atherosclerotic plaque progression by acting against pro-atherogenic oxysterol signaling in M1 and M2 macrophages	[57]
In vitro In vivo	THP-1 monocytes, Mice	0, 25, 50, 100 μM (dose-dependent), 10 mg/kg/day	Ameliorating atherosclerosis partially through restoring intracellular GSH via AMPK-α activation, inhibiting monocyte differentiation, and reducing pro-inflammatory cytokine production	[59]
In vivo	Rats	50 mg/L	Preventing the pathological progression of hypertension through Nrf2 activation	[60]
In vitro; In vivo	Rat aortic smooth muscle cells; Mice	100 μmol/L, ~320 mg/kg	Lowering blood pressure by inducing oxidative activation of cGMP-dependent PKG1α	[61]
In vivo	Rats	50 mg/kg/day	Preventing the activation of inflammasome via downregulating NF-κB p65 and p38 MAPK expression, and upregulating SIRT1 expression	[62]
In vivo	Mice	20 mg/kg	Regulated the FERM-kinase and Nrf2 interaction, decreasing the expression of ICAM-1, and inhibiting monocyte adhesion	[63]
In vivo	Rats	1.24 μg/d	Improving the cardiac and vascular autonomic function	[65]
In vitro	Human RBCs	100 μM	Protecting the erythrocytes via interacting with hemoglobin and reducing heme-iron oxidation	[66]
*Cancers*				
In vitro	LNCaP cells	5, 10, 20, 50 μM	Inducing the expression of COX-2, promoting ERK1/2 activation, and facilitating p53-dependent anti-proliferation gene expression	[14]
In vitro; In vivo	tBregs; Mice	12.5 μM; 20, 50, 500 μg/mouse	Preventing breast cancer metastasis by promoting antitumor immune responses via blunting STAT3, leading to inhibited generation and function of tBregs as well as decreased production of TGF-β	[67]
In vivo	Mice	150, 300 ppm	Inhibiting the formation and growth of colorectal cancer by downregulating oncogenic KRAS expression	[68]
In vitro; In vivo	NSCLC cells Mice	25, 50, 100 μM, 30 mg/kg every 3 days	Preventing tumorigenesis and progression by interrupting glycolysis via inhibition of hexokinase II expression, which was mediated by downregulation of EGFR/Akt/ERK1/2 signaling pathway	[69]
In vitro	MCF-7 cells MVLN cells	Low: 0.1 and 1 μM; High: 10 and 25 μM;	Low concentrations: Increasing the growth of ERα+ cells High concentrations: Inhibiting the proliferation of eERα+ breast cancer	[75]
In vitro	KPL-1, MCF-7, MKL-F cells	Low (KPL-1, ≤22 μM; MCF-7, ≤4 μM); High: ≥44 μM	Low concentrations: Causing cell proliferation ER+ cells High concentrations: Suppressing cell growth	[76]
In vitro In vivo	Apc10.1 cells; Mice; Humans	0.001–1 μM; 0.7, 14.3 mg/kg diet; 5 mg, 1 g	Lower doses of resveratrol: Showing superior efficacy than high doses due to the pro-oxidant activity and AMPK signaling upregulation	[79]
In vitro	A2780, OVCAR-3, SKOV-3 cells	10, 50, 100 μM	Decreasing the efficiency of ovarian cancer cells adhering to peritoneal mesothelium by downregulating the production of α5β1 integrins and upregulating the release of soluble hyaluronic acid	[70]
In vitro	Hela cells	0.1, 1, 10 μM, 10, 20, 50, 100 μM	Inhibiting the expression of PLSCR1, leading to the growth inhibition of HeLa cells	[71]
In vitro	HepG2 cells	25, 50, 100, 200 μM	Inhibiting proliferation and inducing apoptosis by activating caspase-3 and caspase-9, upregulating the Bax/Bcl-2 ratio, and inducing p53 expression	[72]
In vitro	SGC7901 and BGC823 cells	5, 10, 25, 50, 100, 200, and 400 μM	Inhibiting the invasion and migration of human gastric cancer cells by blocking the MALAT1-mediated epithelial-to-mesenchymal transition	[73]
*Liver diseases*				
In vivo	Mice	0.2% of diet	Improving HFD-induced fatty liver by downregulating adipose differentiation-related proteins and increasing the numbers of CD68^+^ Kupffer cells	[9]
In vivo	Rats	10 mg/kg	Attenuating hepatic fibrosis by restoring the architecture and normalizing collagen deposition, mainly due to its antioxidative activities and downregulation of α-SMA	[80]
In vivo	Rats	50, 100 mg/kg	Alleviating NAFLD by upregulating LDLR and SRB1 gene expressions	[83]
In vivo	Rats	250 mg/kg/day	Downregulating HIF-1α expression and mitochondrial ROS production	[85]
In vitro; In vivo	HepG2 cells; Mice	45 μmol 10, 30, 100 mg/kg	Restoring the morphology and function of alcohol-injured liver through inducing autophagy	[86]
In vivo	Rats	10 mg/kg	Mitigating liver cirrhosis by improving the homing of bone marrow-derived mesenchymal stem cells	[87]
*Diabetes*				
In vivo	Rats	20 mg/kg	Increasing insulin action and glucose utilization due to visfatin expression restoration, SIRT1 activation, and glucose transporter modulation	[89]
In vivo	Mice	50 mg/kg	Increasing glucose uptake to improve insulin resistance in the muscle by decreasing DAG accumulation and PKC-θ translocation, and preventing lipolysis under the condition of adipose hypoxia	[90]
In vivo	Rats	147.6 mg/kg/day	Preventing the offspring’s glucose intolerance and islet dysfunction	[91]
In vivo	Mice	0.3% diet	Reducing blood glucose levels, plasma lipids, and free fatty acids, inhibiting the expression of inflammatory mediators both in the aorta and in the blood, by inhibiting the NF-κB pathway	[92]
In vivo	Mice	50 mg/kg	Preventing ROS-mediated mitochondrial fission via AMPK-dependent upregulation of Drp1 phosphorylation, and blocking the activation of NALP3 inflammasome via inhibition of ERS	[93]
*Obesity*				
In vivo	Zebrafish	40 mg/kg/day	Inhibiting transcriptional regulators such as EP300	[95]
In vivo	Mice	0.06% diet	Decreasing the body weight and fat mass, reducing leptin and lipids in plasma, modulating metabolism of glucose and insulin, and restoring immune dysfunction by activating PI3K/SIRT1 and Nrf2 signaling pathway	[96]
In vitro; In vivo	3T3-L1 cells; Mice	0.03 to 100 μM; 1, 10, 30 mg/kg	In vitro: low concentrations of resveratrol (1-10 μM) suppressed adipogenic differentiation in pre-adipocytes, downregulated the expression of PPAR-γ and perilipin protein in differentiated adipocytes, and inhibiting TNF-α-induced lipolysis in mature adipocytes In vivo: Dose-dependently decreasing weight gain and lipid deposition in the liver and adipose tissue	[97]
In vitro	RAW 264.7 macrophage cells	25 μM	Enhancing the catecholamine production, accompanying by suppressing the pro-inflammatory M1 macrophages, and activating anti-inflammatory M2 macrophages in white adipose tissue	[98]
In vivo	Mice	0.2% diet	Promoting white adipose browning and thermogenesis in the male descendants, and these health benefits persisted and prevented obesity in their future life	[99]
In vitro; In vivo	L6 myogenic cell line; Rats	1, 5, 10, 25 or 50 μM; 0.4% diet	In vitro: Improving mitochondrial function and reducing oxidative stress through the PKA/LKB1/AMPK pathway; In vivo: Preventing muscle loss and myofiber size decrease, improving grip strength, and abolishing excessive fat accumulation	[100]
In vivo	Mice	0.06% diet	Improving obesity-related complications by restoring plasma thyroid hormone levels, and attenuating oxidative stress in the heart	[101]
In vitro	Human sperm	2.6, 6, 15, 30, 50, 100 μmol/L	Improving obesity-related complications by restoring reproductive dysfunction like infertility	[102]
*Alzheimer’s disease and Parkinson’s disease*				
In vivo	Rats	20 mg/kg/day	Ameliorating ERS by downregulating the gene expression of CHOP and GRP78, inhibiting caspase-3 activity, and ameliorating oxidative damage via suppressing xanthine oxidase activity and protein carbonyl formation as well as activating glutathione peroxidase and Nrf2 signaling pathway	[10]
In vitro	CL2006 cells	100 μM	Inhibiting the aggregation of Aβ by modulating specific proteins such as UBL/XBP-1 involved in proteostasis	[103]
In vivo	Mice	16 mg/kg/day	Preventing memory loss by decreasing elevated levels of mitochondrial complex IV protein in the mouse brain via the activation of SIRT1 and AMPK pathways	[104]
In vivo	Mice	100 mg/kg/day	Preventing memory loss via the activation of SIRT1 and AMPK pathways	[105]
In vitro; In vivo	SH-SY5Y cells; Mice	50 μM; 50 mg/kg	Elevating miR-214 expression, leading to decreased mRNA expression of α-synuclein	[106]
*Sex-dependent effects of resveratrol*				
In vivo	Rats	2.5 mg/kg/day	Superior improvements of MI in females in terms of IVSDs, ESV, EF, FS, and IVRT, among which IVRT is purely sex-dependent	[109]
In vivo	Rats	50 mg/L in drinking water	Increasing the relaxations to estrogen in aortae, more potent in males, probably due to resveratrol’s promoting nitric oxide and/or suppressing superoxide effects	[110]
In vitro; In vivo	MESC2.10 and SN4741 cells; Mice	20 mg/kg; 10 μM	Increasing DAT in the striatum in females but not in males; Upregulating DAT in the dopaminergic cells by inducing its gene transcription	[111]
In vivo	Mice	100 mg/kg	Adverse effects in females but not in males, regarding weight loss, stool consistency, and discomfort	[112]

Abbreviations used in the table: AC, acetyl; AhR, aryl hydrocarbon receptor; Akt, protein Kinase B; AMPK, AMP-activated protein kinase; Aβ, amyloid β; cAMP, cyclic adenosine monophosphate; CAT, catalase; cGMP, cyclic guanosine monophosphate; CHOP, C/EBP homologous protein; COX-2, cyclooxygenase-2; DAG, diacylglycerol; DAT, dopamine transporter; EF, ejection fraction; EGFR, epidermal growth factor receptor; eNOS, endothelial nitric oxide synthase; ERK, extracellular signal-regulated kinases; ERRα, estrogen related receptor α; ERS, endoplasmic reticulum stress; Erα, estrogen receptor α; ERα+, estrogen receptor alpha positive; ESV, end systolic volume; EV71, enterovirus 71; FERM, band 4.1, ezrin, radixin, and moesin; FoxO1, forkhead box protein O1; FS, fractional shortening; GPx, glutathione peroxidase; GRP78, glucose-regulated protein 78; GβL, G protein beta subunit-like; HFD, high-fat diet; HIF-1α, hypoxia-inducible factor 1α; HMGB1, high mobility group box 1; HMGB1, high mobility group box 1; HO-1, heme oxygenase (decycling) 1; HSL, hormone-sensitive lipase; ICAM-1, intercellular adhesion molecule-1; IFN-γ, interferon γ; IKK, IκB kinase; IL-1β, interleukin-1β; IVRT, isovolumic relaxation time; IVSDs, interventricular septal wall dimension at systole; IκBα, nuclear factor of kappa light polypeptide gene enhancer in B-cells inhibitor α; JAK, Janus kinase; Keap1, Kelch-like ECH-associated protein 1; LDLR, low-density lipoprotein receptor; LKB1, liver kinase B1; LPS, lipopolysaccharides; MALAT1, metastasis-associated lung adenocarcinoma transcript 1; MAP2K, mitogen-activated protein kinase kinase; MAPK, mitogen-activated protein kinase; MCP-1, monocyte chemoattractant protein-1; MI, myocardial infarction; mSIN1, mammalian stress-activated protein kinase interacting protein 1; mTOR, mammalian target of rapamycin; mTORC2, mTOR Complex 2; NAD, nicotinamide adenine dinucleotide; NAFLD, non-alcoholic fatty liver disease; NALP3, NACHT, LRR, and PYD domains-containing protein 3; NF-κB, nuclear factor kappa-light-chain-enhancer of activated B cells; Nrf2, nuclear factor (erythroid-derived 2)-like 2; p53, phosphoprotein p53; PDE 3B, phosphodiesterase 3B expression; PDK1, phosphoinositide dependent kinase 1; PGC, peroxisome proliferator-activated receptor gamma coactivator 1α; PI3K, phosphatidylinositol 3-kinase; PIP2, phosphatidylinositol 4,5-bisphosphate; PIP3, phosphatidylinositol-3,4,5--trisphosphate; PKA, protein kinase A; PKC-θ, protein kinase C θ; PKG1α, cGMP-dependent protein kinase 1α; PLSCR1, phospholipid scramblase 1; PPAR-γ, peroxisome proliferator-activated receptor γ; PTEN, phosphatase and tensin homolog; RANTES, regulated on activation normal T cell expressed and secreted; RICTOR, the rapamycin-insensitive companion of mTOR; SARM, sterile α and armadillo motif protein; SIRT, sirtuin 1; α-SMA, smooth muscle actin; SOD, superoxide dismutase; SRB1, scavenger receptor class B type I; STAT, signal transducer and activator of transcription; tBregs, tumor-evoked regulatory B cells; TF, transcription factor; TGF-β, transforming growth factor β; TLR4, toll-like receptor 4; TNF-α, tumor necrosis factor α; TRIF, toll/IL-1 receptor domain-containing adaptor inducing β interferon; UBL, ubiquitin-like protein; XBP-1, X-box binding protein 1.

## 4. Clinical Trials

In light of the positive epidemiological evidence and the promising results from experimental studies, resveratrol has been investigated in the human population as a potential nutraceutical (Table 3). There are certain encouraging outcomes reported. Specifically, resveratrol intake (500 mg/day for 30 days) was demonstrated to reduce CVD risk factors by increasing SIRT1, enhancing total antioxidant capacity in healthy individuals, decreasing low-density lipoprotein cholesterol (LDL-C), ApoB, and oxidized LDL [113,114,115]. Moreover, resveratrol prevented bone density loss (500 mg/day for 6 months) in type-2 diabetic patients [116]. In addition, resveratrol showed benefits in obesity, NAFLD, and neurodegenerative diseases [15,16,117,118]. However, some null outcomes have also been reported. For instance, resveratrol intake (250 mg/day for 8 weeks) did not increase SIRT1 nor improve many cardiovascular risk factors in healthy aged men [58]. In some other studies, no significant improvements were found in metabolic biomarkers in patients with Alzheimer’s disease, obesity or type-2 diabetes, respectively, though their resveratrol intake ranged from 150 to 1000 mg/day with different duration of 4–52 weeks [119,120,121,122]. Therefore, the outcomes of clinical studies are not always consistent. Of note, the health effects of resveratrol as a therapeutic intervention may be affected by many factors, such as baseline health status of the subjects, their demographic profile, lifestyle, eating pattern, resveratrol dose, and intervention period. Nevertheless, a well-designed study, proper sample size, and a scientific evaluation system are also needed. Furthermore, although resveratrol is well-tolerated and safe as reported by most of the clinical trials, very few adverse effects (e.g., nausea and diarrhea) were observed, as well as some unfavorable results like an increase in total cholesterol, ApoB, the homeostatic model assessment-insulin resistance (HOMA-IR) score, fasting blood glucose, body fat, and the inflammatory markers [15,114,118]. Interestingly, resveratrol was reported to mask the exercise training-induced benefits, blunting the improved cardiovascular health parameters [58]. It might be attributable to the potent antioxidant capability of resveratrol, which could scavenge the free radicals induced by exercise training, because the appropriate number of free radicals is necessary for health maintenance. Therefore, it could be suggested that foods containing resveratrol should not be consumed during exercise.

## 5. Conclusions

Resveratrol is one of the most investigated bioactive compounds in foods. A number of epidemiologic studies have demonstrated that resveratrol is effective in the prevention of some diseases such as CVDs and cancer, although the results are sometimes inconsistent. In addition, the experimental studies have shown that resveratrol possesses many bioactivities and health benefits like antioxidant, anti-inflammatory, immunomodulatory effects, and improving CVDs, cancer, liver diseases, diabetes, obesity, Alzheimer’s disease, and Parkinson’s disease. Furthermore, resveratrol showed some effects in patients with CVDs and obesity in clinical trials, although inconsistency has also been reported. In the future, more bioactivities and health benefits of resveratrol should be evaluated, and further clarification of the underlying mechanisms of action is required. In order to develop resveratrol into functional foods and pharmaceuticals, more clinical trials are essential to confirm its efficacy and observe the possible adverse events, and the dose–effect relationship should be paid special attention as well. 

## Figures and Tables

**Table 1:** The results of resveratrol from observational studies.

Population/Country	Study Name/Type	Sample Size (Valid Data)	Dose and Schedule	Main Findings: Resveratrol vs. Measurements/Risk Factors/Biomarkers	Ref.
Swiss/Switzerland	Case-control	N = 971 (all female; case, 369; control, 602)	Tertiles: T1: 0.0–73.0 μg/day T2: 73.1–160.7 μg/day T3: >160.7 μg/day (food-frequency questionnaire, FFQ, on weekly frequency, 2 years prior)	***Favorable:*** Inversely associated with breast cancer risk (OR (95% CI): T2 vs. T1, 0.64 (0.44–0.93); T3 vs. T1, 0.55 (0.39–0.76))	[17]
Iranian (Tehranian)/Iran	Cross-sectional study, part of the TLG study	N = 2618 (male, 1162; female, 1456)	Quartiles: Q1: 0.014 mg/day Q2: 0.015–0.027 mg/day Q3: 0.028–0.053 mg/day Q4: 0.054 mg/day (FFQ on a daily frequency, 1 year prior)	***Null:*** Significantly associated with WC, TG, HDL, BG, and MS ***Unfavorable:*** The top quantile of intake (0.054 mg/day and more) was positively associated with high BP (HR = 1.52; 95% CI: 1.02–2.27)	[19]
Spanish/Spain	Cross-sectional study, part of the PREDIMED study	N = 1000 (male, 479; female, 521)	Quintiles: Q1: 0.48 mg/day Q2: 1.04 mg/day Q3: 2.04 mg/day Q4: 5.75 mg/day (FFQ, 1 year prior)	***Favorable:*** Significantly decreased CVD risk factors (FBG (95% CI: −1.033 to −0.033); TG (95% CI: −1.998 to −0.029); and heart rate (95% CI: −0.467 to −0.087)). ***Null:*** Resveratrol intake was not significantly associated with TC, HDL, LDL and BP	[6]
Spanish/Spain	Cross-sectional study, part of the PREDIMED study	N = 7172 (male, 3249; female, 3923)	Quintiles: Q1: 0.48 mg/d Q2: 1.04 mg/d Q3: 2.04 mg/day Q4: 5.75 mg/day (FFQ, 1 year prior)	***Favorable:*** High dose intake (5.75 mg/d) significantly reduced all-cause mortality by 52% (HR = 0.48; 95% CI: 0.25–0.91) ***Null:*** No significant CVD risk reduction (HR = 0.77; 95% CI: 0.35–1.72)	[21,22]
Swedish/Sweden	Case-control study	N = 1400 (case, 594 including (OAC, 181; OSCC, 158; JAC, 255)) (control, 806)	Control: 0.1 mg/day OAC: 0.07 mg/day OSCC: 0.11 mg/day JAC: 0.09 mg/day (FFQ, 20 years prior)	***Favorable:*** In a significantly negative association with the risk of 3 subtypes of esophageal cancer (OAC (95% CI: 0.12–0.49); OSCC (95% CI: 0.15–0.65), and JAC (95% CI: 0.28–0.84))	[5]
Italian/Italy	Cohort study, “Aging in the Chianti Region”	N = 529 (male, 236; female, 293)	Tertiles: T1: 0.1 mg/day T2: 0.1–1.1 mg/day T3: >1.1 mg/day (FFQ)	***Favorable:*** Inversely associated with the risk of frailty syndrome during the first 3-year follow-up (T3 vs. T1: OR = 0.11; 95% CI: 0.03–0.45) ***Null:*** No substantial association with (i) risk of frailty syndrome in 6-, or 9-year follow-up; (ii) inflammatory biomarkers including IL-6, IL-1β, TNF-α, and CRP; (iii) CVD, cancer or all-cause mortality	[18]
Chinese/China	Cross-sectional study,	N = 1393 (male, 446; female, 947)	Mean: 0.15 mg/d (FFQ, 1 year prior)	***Null:*** Not significantly associated with CVD risk factors including BP, BG, lipid profiles (TC, TG, HDL, and LDL), and carotid IMT	[23]

Abbreviations used in the table: BG, blood glucose; BP, blood pressure; CI, confidence intervals; CRP, C-reactive protein; FBG, fasting blood glucose; FFQ, food-frequency questionnaire; HDL, high-density lipoprotein; HR, hazard ratios; IL, interleukin; IMT, intima–media thickness; JAC, (gastro-esophageal) junctional adenocarcinoma; LDL, low-density lipoprotein; MORGEN study, Monitoring Project on Risk Factors and Health in the Netherlands study; MS, metabolic syndrome; OAC, esophageal adenocarcinoma; OR: odds ratio; OSCC, esophageal squamous-cell carcinoma; PREDIMED study: Prevención con Dieta Meniterránea study; TC, total cholesterol; TG, triglyceride; TLGS: Tehran lipid and glucose study; TNF-α, tumor necrosis factor α; WC, waist circumference.

**Table 3:** The results of resveratrol from clinical research.

Population	Targeting Diseases	Study Type	Sample Size (Valid Data)	Resveratrol Dose and Duration	Main Findings: Resveratrol vs. Measurements/Risk Factors/Biomarkers	Ref.
Healthy and slightly overweight	CVD—atherosclerosis	Randomized, parallel	N = 48 (male, 24; female, 24)	Resveratrol supplement, 500 mg/day (30 days)	***Favorable:*** Increased serum SIRT1 concentrations from 1.06 ± 0.71 to 5.75 ± 2.98 ng/mL, *p* < 0.0001 ***Null:*** Did not influence the various metabolic parameters (BW, BMI, WC, HR, BP, HDL, LDL, TG, BG, estradiol, estrone, insulin, hsCRP, and TAC) Unfavorable: Increased TC, ApoB, and HOMA-IR score	[114]
Asymptomatic hypercholesterolemics (AHCs) and normohypercholemics (NC)	CVD—atherogenesis	Randomized, placebo-controlled	N = 40 (male, 21; female, 19)	Resveratrol supplement, 150 mg/day (4 weeks)	***Favorable:*** Increased TAC (mean value increased to 136.7% after consumption, *p* = 0.035) in healthy NC individuals and facilitated an increase in vitamin E (7.18 μmol/l, i.e., 35.72%) in AHC ***Null:*** No differences found in TC, TG, HDL, LDL, TAC (in AHC), and vitamin E (in NC)	[113]
Overweight and slightly obese volunteers	CVD—endothelial function	Randomized, double-blind, placebo-controlled	N = 45 (male, 25; female, 20)	Trans-resveratrol supplement,150 mg/day (4 weeks)	***Null:*** Did not improve endothelial function (FMD, arterial stiffness, and other endothelial activation markers), inflammation (IL-6 and TNF-α), glucose and lipid metabolism (BG, insulin, and serum TG)	[122]
65 years or older with peripheral artery disease (PAD)	CVD—PAD	Randomized, double-blind, placebo-controlled	N = 66 (male, 45; female, 21)	Trans-resveratrol supplement,125 and 500 mg/day (6 months)	***Favorable:*** 125 mg/day improved the outcome of 6-min walk test results statistically significant (95% CI: −5.7 to 39.5) but not clinically meaningful ***Null:*** 500 mg/day showed no significant improvement	[123]
Patients in primary cardiovascular disease prevention	CVD—atherogenesis	Triple-blind, randomized, placebo-controlled	N = 75 (male, 34; female, 41)	Resveratrol-enriched grape extract, 350 mg/day (6 months)	***Favorable:*** Decreased LDL-C (−4.5%, *p* = 0.04), ApoB (−9.8%, *p* = 0.014), oxidized LDL (−20%, *p* = 0.001), and oxidized LDL/ApoB (−12.5%, *p* = 0.000); increased ratio non-HDL-C/ApoB (8.5%, *p* = 0.046) ***Null:*** No clinically significant effects on hepatic, thyroid, and renal function (GGT, AST, ALP, bilirubin and albumin; TSH, T4; CPK, creatinine, and urate)	[115]
Healthy aged men	CVD	Randomized, double-blind, placebo-controlled	N = 27 (male)	Trans-resveratrol supplement, 250 mg/day (8 weeks)	***Null:*** No effects on SIRT1 protein concentrations or cardiovascular parameters (BG, TC, and HDL), and VCAM-1 ***Unfavorable:*** Abolished the exercise training-induced improvement in maximal oxygen uptake, BP, and lipids (LDL, TC/HDL ratio, and TG)	[58]
Women at increased breast cancer risk	Cancer—breast cancer	Randomized, double-blind, placebo-controlled	N = 39 (male)	Trans-resveratrol supplement, 10 or 100 mg/day (12 weeks)	***Favorable:*** Decreased the methylation of RASSF-1α (*p* = 0.047) ***Null:*** Did not significantly alter PGE2	[124]
Patients with type-2 diabetes	Type 2 diabetes	Randomized, double-blind, placebo-controlled	N = 192 (male, 126; female, 66)	Resveratrol supplement, 40 and 500 mg/day (6 months)	***Favorable:*** Prevented bone density loss (500 mg/d) (whole-body BMD (0.01 vs. −0.03 g/cm^2^, *p* = 0.001), whole-body BMC (4.04 vs. −58.8 g, *p* < 0.001), whole-body T-score (0.15 vs. −0.26), and serum phosphorus (0.07 vs. −0.01 μmol/L, *p* = 0.002)); decreased CRP (not significantly) ***Null:*** BW, BMI, WC, BP, FBG, HbA1c, insulin, HOMA-IR, C-peptide, FFAs, ALT, AST, GGT, uric acid, IL-6, and adiponectin ***Unfavorable:*** Slightly increased TC and TG (500 mg/d)	[116]
Patients with diet-controlled type-2 diabetes	Type 2 diabetes	Randomized, double-blind, placebo-controlled	N = 14 (male)	Resveratrol capsules, 1000 mg/day (5 weeks)	***Favorable:*** Modestly decreased FBG and HbA1c ***Null:*** No significant effects on GLP-1 secretion, gastric emptying, glycemic control (HbA1c, BG), energy intake, and BW	[121]
Obese men	Obesity	Randomized, placebo-controlled	N = 24 (male)	Trans-resveratrol tablets, 500 mg/day (4 weeks)	***Null:*** Did not improve insulin sensitivity, BP, BG, insulin, HOMA-IR, HbA1c, lipids (TC, HDL-C, LDL-C and TG), liver and skeletal muscle lipid content, or inflammatory biomarkers (IL-6, TNF-α, and MCP1) and some metabolic markers Unfavorable: Insignificantly deteriorated insulin sensitivity	[120]
Overweight/obese with insulin-resistance subjects	Obesity	Randomized, double-blind, placebo-controlled	N = 108 (male, 54; female, 54)	Resveratrol supplement, 150 mg/day (12 weeks)	***Null:*** Did not significantly impact liver fat content or cardiometabolic risk biomarkers (FBG, HbA1c, BP, TC, HDL, LDL, TG, AST, ALT, GGT, hsCRP, and IL-6)	[119]
Overweight/obese with NAFLD	Obesity—NAFLD	Randomized, placebo-controlled	N = 75 (male, 52; female, 23)	Resveratrol capsules, 600 mg/day (12 weeks)	***Favorable:*** Significantly reduced BW (95% CI: −1.61 to −0.38) and BMI (95% CI: −0.54 to −0.12) ***Null:*** Did not significantly change ALT, AST, and lipid profiles (TG, TC, LDL-C, HDL-C), hepatic steatosis grade (ALT and AST), serum glycemic parameters (FBG, insulin, and HbA1c), and SIRT1 levels	[117]
Obese men	Obesity—bone health	Randomized, double-blind, placebo-controlled	N = 66 (male)	Trans-resveratrol tablets, 1000 or 150 mg/day, (16 weeks)	***Favorable:*** Dose-dependently benefited bone by stimulating formation or mineralization as significantly increased BAP (R = 0.471, *p* < 0.001), and BMD (BAP and lumbar spine volumetric BMD were positively correlated: R = 0.281, *p* = 0.027)	[16]
Individuals with mild/moderate Alzheimer disease (AD)	Aging—AD	Randomized, double-blind, placebo-controlled	N = 119 (male, 51; female, 68)	Resveratrol supplement, 500–2000 mg/day (52 weeks)	***Uncertain:*** Resveratrol and its major metabolites were detectable in plasma and CSF, suggesting CNS effects; CSF Aβ40 and plasma Aβ40 levels declined less than those in the placebo group; brain volume loss was more compared to placebo ***Null:*** No effects on plasma Aβ42, CSF Aβ42, CSF tau, CSF phospho-tau 181, hippocampal volume, entorhinal cortex thickness, MMSE, CDR, ADAS-cog, NPI, or glucose and insulin metabolism ***Unfavorable:*** The most common adverse events were nausea and diarrhea, but similar to placebo	[118]
Elderly participants	Aging—memory	Randomized, double-blind, placebo-controlled	N = 53 (male, 25; female, 28)	Resveratrol pills, 200 mg/day, (26 weeks)	***Favorable:*** Non-significant trend for stable performance in a pattern recognition task ***Null:*** No significant changes in CVLT performance, HbA1c levels, hippocampus volume, microstructure, and functional connectivity ***Unfavorable:*** Increased serum cholesterol, weight, body fat, FBG, and inflammatory markers; decreased physical activity and neurotrophic factors ***Adverse events:*** Two dropouts (a sudden decrease in eyesight and a skin rash), and others (case no.): Diarrhea (*n* = 3), skin changes (*n* = 3), stomach aches (*n* = 1), dizziness (*n* = 1), improved mood changes (*n* = 1), loss of hair (*n* = 1)	[15]

Abbreviations used in the table: ADAS-cog, Alzheimer’s Disease Assessment Scale–cognitive; AHC, asymptomatic hypercholesterolemics; ALP, alkaline phosphatase; ALT, aminotransferase; ApoB, apolipoprotein B; AST, aminotransferase; Aβ40, amyloid β40; BAP, bone alkaline phosphatase; BG, blood glucose; BMC, bone mineral content; BMD, bone mineral density; BMI, body mass index; BP, blood pressure; BW, body weight; CDR, clinical dementia rating; CNS, central nervous system; CPK, creatine phosphokinase; CRP, C-reactive protein; CSF, cerebrospinal fluid; CVLT, California Verbal Learning Task; CVR, cerebrovascular responsiveness; FBG, fasting blood glucose; FFAs, free fatty acids; FMD, flow-mediated vasodilation; GGT, γ-glutamyl transferase; GLP-1, glucagon-like peptide 1; HbA1c, glycated hemoglobin; HDL, high-density lipoprotein; HOMA-IR, the homeostatic model assessment—insulin resistance; HR, heart rate; hsCRP, high-sensitivity C-reactive protein; IL-6, interleukin-6; LDL-C, low-density lipoprotein cholesterol; MCP1, monocyte chemoattractant protein 1; MMSE, mini-mental state examination; NAFLD, non-alcoholic fatty liver disease; NC, normohypercholemics; NPI, neuropsychiatric inventory; PAD, peripheral artery disease; PGE2, prostaglandin E2; SIRT1, sirtuin 1; T4, thyroxine; TAC, total antioxidant capacity; TC, total cholesterol; TG, triglycerides; TNF-α, tumor necrosis factor α; TSH, thyroid-stimulating hormone; VCAM-1, vascular cell adhesion molecule 1; WC, waist circumference; WHR, waist-hip ratio.
